# Incidental Combined Hepatocellular-Cholangiocarcinoma in Liver Transplant Recipients: A Matched Cohort Study

**DOI:** 10.3389/ti.2025.15298

**Published:** 2026-01-28

**Authors:** Sudha Kodali, Ashton A. Connor, David W. Victor, Maen Abdelrahim, Ahmed Elaileh, Khush Patel, Elizabeth W. Brombosz, Edward A. Graviss, Duc T. Nguyen, Susan Xu, Linda W. Moore, Mary R. Schwartz, Sadhna Dhingra, Tamneet Basra, Michelle R. Jones-Pauley, Mazen Noureddin, Constance M. Mobley, Mark J. Hobeika, Caroline J. Simon, Yee Lee Cheah, Kirk Heyne, Ahmed O. Kaseb, Ashish Saharia, A. Osama Gaber, R. Mark Ghobrial

**Affiliations:** 1 Sherrie and Alan Conover Center for Liver Disease and Transplantation, Houston Methodist Hospital, Houston, TX, United States; 2 JC Walter Jr Transplant Center, Houston Methodist Hospital, Houston, TX, United States; 3 Department of Medicine, Houston Methodist Hospital, Houston, TX, United States; 4 Department of Medicine, Weill Cornell Medical College, New York, NY, United States; 5 Department of Surgery, Houston Methodist Hospital, Houston, TX, United States; 6 Department of Surgery, Weill Cornell Medical College, New York, NY, United States; 7 Department of Oncology, Neal Cancer Center, Houston Methodist Hospital, Houston, TX, United States; 8 Department of Pathology and Genomic Medicine, Houston Methodist Hospital, Houston, TX, United States; 9 Department of Pediatrics, Baylor College of Medicine, Houston, TX, United States; 10 Center for Health Data Science and Analytics, Houston Methodist Hospital, Houston, TX, United States; 11 Houston Research Institute, Houston, TX, United States; 12 Department of Gastrointestinal Medical Oncology, Division of Cancer Medicine, University of Texas MD Anderson Cancer Center, Houston, TX, United States

**Keywords:** hepatocellular carcinoma, cholangiocarcinoma, liver transplantation, transplant oncology, liver neoplasms

## Abstract

Mixed hepatocellular carcinoma (HCC) with cholangiocarcinoma (HCC-CCA) is an aggressive primary liver cancer and difficult to distinguish from HCC using non-invasive methods. Outcomes of patients incidentally diagnosed with HCC-CCA after LT relative to pure HCC with similar tumor burden were investigated. Medical records of patients undergoing LT (n = 1,898) for HCC (n = 493) from 6/2008–9/2023 were reviewed. Patients incidentally diagnosed with HCC-CCA were propensity matched to HCC patients undergoing LT. Independent analyses were performed using pre-LT (Match1; identifiable pre-LT) and explant pathology (Match2, more prognostic) characteristics. Incidental HCC-CCA occurred in 19 (3.9%) patients; all assumed to have HCC pre-LT and received HCC-directed neoadjuvant treatment. When matched on pre-LT characteristics (Match1, n = 57), more patients with HCC-CCA were outside Milan or University of California, San Francisco criteria on explant (p = 0.01). More patients with HCC-CCA underwent neoadjuvant microwave ablation (p = 0.02) compared to HCC Match2 (n = 45) but were otherwise similar demographically and clinically. Overall and recurrence-free survival were lower for HCC-CCA in Match1 (p = 0.003 and p < 0.001, respectively) and Match2 (p < 0.001 and p = 0.001, respectively). HCC-CCA has an aggressive phenotype with high recurrence after LT. Better screening tools and biomarkers are needed to distinguish HCC-CCA from HCC to ensure patients receive appropriate treatment and maximize post-LT outcomes.

## Graphical Abstract

**Figure:**
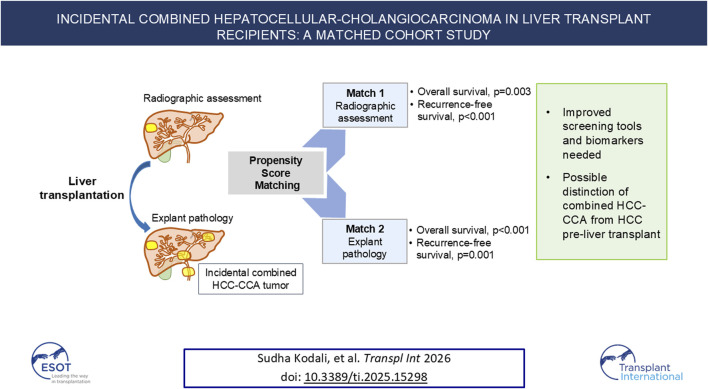

## Introduction

Primary hepatic malignancies are increasing in incidence and are now the third leading cause of cancer-related deaths worldwide [1]. Combined or mixed hepatocellular carcinoma-cholangiocarcinoma (HCC-CCA), accounting for around 0.4%–14.2% of primary liver cancers, is rare and often misclassified as HCC pre-transplant with worse outcomes [2]. Studies indicate that HCC-CCA tumors are more aggressive than HCC tumors and are associated with poorer prognosis than either HCC and or CCA alone [2–5].

Unlike HCC, HCC-CCA has largely been considered a contraindication for liver transplantation (LT) due to the increased risk of post-transplant recurrence and poor outcomes [6]. LT does provide a survival benefit over resection in patients with mixed tumors [7–9], but that benefit has traditionally been outweighed by the need to pursue utility in deceased donor grafts allocation. Importantly, most HCC-CCA cases in LT recipients were considered to be HCC alone prior to transplantation due to the difficulty in distinguishing HCC-CCA from HCC radiologically [10–13]. Thus, incidental diagnosis seems to be the norm for patients with HCC-CCA undergoing LT.

Some reports indicate an increasing incidence of incidental HCC-CCA in LT recipients in recent years [8, 10]. It is important to determine optimal treatment regimens to provide the best medical care possible to patients with HCC-CCA. Given the rarity of this type of tumor and that LT is not standard-of-care, a detailed description of the trajectory of patients with HCC-CCA provides important information on clinical outcomes. The primary aim of this paper is to describe the outcomes of LT recipients presumed to have HCC alone pre-LT and received neoadjuvant treatment for HCC but found to have HCC-CCA on explant. The secondary aims were to compare pre-LT (representing clinical decision making) versus explant predictors and to examine the patterns of recurrence and treatment.

## Materials and Methods

Medical records of 1,898 adult patients undergoing LT at a single, quaternary care institution between June 2008 and September 2023 were reviewed. Patients diagnosed with mixed HCC-CCA on explant were included in the primary analysis. All work was carried out with approval from the Houston Methodist Research Institute Institutional Review Board under protocol number Pro00000587 with a waiver of authorization. The center follows the guidance of the Declaration of Istanbul on Organ Trafficking and Transplant Tourism.

A multidisciplinary tumor board reviewed the medical records of patients referred for LT who had a diagnosis of liver cancer and made clinical care recommendations, including systemic therapy and locoregional therapy (LRT). Patients with large, single tumors (>5 cm in diameter), multifocal lesions, or poorly differentiated tumors received combined neoadjuvant systemic therapy and LRT. Neoadjuvant treatment was HCC-directed, as all patients with HCC-CCA were believed to have HCC prior to undergoing LT. All patients in the study were appropriately treated and down staged with LRT. Microwave ablation, TACE, and TARE were the LRT modalities utilized. Decisions to place patients on the LT waitlist were made by a multidisciplinary transplant medical review board. While on the LT waitlist, patients underwent close monitoring, including cross-sectional imaging every 3 months to rule out disease progression.

### Statistical Analysis

HCC-CCA recipients were matched with HCC alone recipients using a propensity score method of 1:3 (as 1 HCC-CCA case to 3 HCC cases), using a non-replacement, caliper width 0.2 approach. Propensity score matching allowed comparisons between patients with similar disease burden. Since the patients with HCC-CCA were determined at the time of liver explant, it was decided to perform 2 independent propensity score matches. Variables were chosen based on established prognostic factors in HCC. The first match utilized characteristics measurable pre-transplant that could inform clinical decision making and transplant candidate selection (“pre-LT match”): pre-transplant alpha-fetoprotein (AFP) levels and total radiologic tumor diameter. No imputation of missing variables was planned.

The second, independently performed propensity score match used features from explant pathology (“explant match”), which are frequently more prognostic than pre-transplant variables in HCC [14–16]. This match more reflects the actual pathologic risk of the lesions on explant. The propensity match was performed using a 1:3 ratio of 1 HCC-CCA to 3 HCC alone cases, again using the non-replacement, 0.2 caliper width approach. The scoring was based on pathologic total tumor diameter, tumor differentiation, and presence or absence of vascular invasion. No imputation of missing variables was planned.

Demographic and clinical data are reported as frequencies and proportions for categorical variables and as median and interquartile range (IQR) for continuous variables. Differences between patients with HCC-CCA and matched patients with HCC were compared using the chi-square or Fisher’s exact tests for categorical variables and Kruskal-Wallis test for continuous variables. Balance of the covariates used as the matching criteria was evaluated by the percent standardized bias. Overall all-cause patient and recurrence-free survival are presented by Kaplan-Meier curves. Differences in survival across groups were compared using the log-rank test. All analyses were performed on Stata version 17.0 (StataCorp LLC, College Station, TX). A P < 0.05 was considered statistically significant.

## Results

### Patients With Mixed Hepatocellular Carcinoma and Cholangiocarcinoma

Of 493 patients having LT for HCC, 19 (3.9%) patients with incidentally diagnosed HCC-CCA underwent LT for HCC during the study period (Table 1). Recipients were predominantly male (15, 78.9%) and white (14, 73.7%). Most had viral disease etiology (Hepatitis C: 8 [42.1%]; Hepatitis B: 2 [10.5%]). All received a deceased donor LT. Median laboratory Model for End-Stage Liver Disease (MELD) score at transplant was 17 (IQR, 9–29). These patients generally had low serum tumor markers: median alpha fetoprotein (AFP) was 7.1 (2.7–22.2) ng/mL at listing and was 5.2 (2.6–20.9) ng/mL at transplant. Median carbohydrate antigen 19–9 (CA19-9) level measured soonest prior to LT was 34.0 (19.3–54.0) U/mL, and 11 (57.9%) patients had “normal” CA19-9 values (<37 U/mL). Only one patient (5.3%) had CA19-9 >100 U/mL.

**TABLE 1:** Clinical characteristics of liver transplant recipients with mixed hepatocellular carcinoma-cholangiocarcinoma and propensity-matched patients with hepatocellular carcinoma only based on pre-transplant variables (“pre-LT match”).

Recipient Characteristics	Mixed tumor	HCC Pre-LT match	p-value
	N = 19	N = 57	
Age (years), median (IQR)	65.2 (61.1, 69.5)	62.0 (57.0, 67.0)	0.12
Sex, n (%)	​	​	0.77
Male	4 (21.1)	15 (26.3)	​
Female	15 (78.9)	42 (73.7)	​
Race/ethnicity, n (%)	​	​	0.76
White	14 (73.7)	39 (68.4)	​
Black	0 (0.0)	4 (7.0)	​
Hispanic	3 (15.8)	10 (17.5)	​
Asian	2 (10.5)	4 (7.0)	​
BMI at LT (kg/m^2^), median (IQR)	29.6 (24.8, 33.9)	27.3 (23.9, 32.7)	0.64
Laboratory MELD at transplant, median (IQR)	17.0 (9.0, 29.0)	13.0 (10.0, 19.0)	0.57
Underlying etiology of liver disease, n (%)	​	​	0.19
Hepatitis C	8 (42.1)	39 (68.4)	​
Hepatitis B	2 (10.5)	3 (5.3)	​
Alcohol-associated liver disease	3 (15.8)	7 (12.3)	​
MASLD or cryptogenic cirrhosis	5 (26.3)	7 (12.3)	​
Other	1 (5.3)	1 (1.8)	​
Waiting time from listing (days), median (IQR)	332.0 (148.0, 806.0)	346.0 (190.0, 525.0)	0.67
Pre-transplant tumor markers			
Last AFP prior to transplant (ng/mL), median (IQR)	5.2 (2.6, 20.9)	6.6 (3.6, 27.3)	0.63
Neutrophil/lymphocyte ratio pre-LT, median (IQR)	3.5 (2.0, 7.2)	5.8 (2.7, 17.9)	0.15
Neoadjuvant therapy			
Neoadjuvant therapy, n (%)			
TACE	11 (57.9)	43 (75.4)	0.16
Radiofrequency ablation	4 (21.1)	13 (22.8)	1.00
Resection	0 (0.0)	2 (3.5)	1.00
Sorafenib	5 (26.3)	14 (24.6)	1.00
Yttrium-90	2 (10.5)	3 (5.3)	0.59
Microwave ablation	3 (15.8)	0 (0.0)	**0.01**
Total number of LRT, median (IQR)	2.0 (1.0, 3.0)	1.0 (1.0, 2.0)	0.09
Pre-transplant radiographic tumor characteristics			
Tumor burden classification, n (%)	​	​	0.54
Within Milan	16 (84.2)	45 (78.9)	​
Outside Milan, within UCSF	2 (10.5)	4 (7.0)	​
Outside UCSF	1 (5.3)	8 (14.0)	​
Pathologic tumor characteristics			
Tumor burden classification, n (%)	​	​	**0.01**
Within Milan	7 (36.8)	38 (66.7)	​
Outside Milan, within UCSF	6 (31.6)	4 (7.0)	​
Outside UCSF	6 (31.6)	15 (26.3)	​
Tumor T stage, n (%)	​	​	0.30
T0	1 (5.3)	1 (2.8)	​
T1s	1 (5.3)	0 (0.0)	​
T1	4 (21.1)	15 (41.7)	​
T2	9 (47.4)	17 (47.2)	​
T3a	2 (10.5)	2 (5.6)	​
T3b	1 (5.3)	1 (2.8)	​
T4	1 (5.3)	0 (0.0)	​
Tumor N Stage, n (%)	​	​	0.37
N0	8 (42.1)	21 (58.3)	​
N1	0 (0.0)	1 (2.8)	​
NX	11 (57.9)	14 (38.9)	​
Microvascular invasion, n (%)	2 (10.5)	2 (5.6)	0.60
Total number of tumors (pathology), median (IQR)	2.0 (2.0, 4.0)	2.0 (1.0, 3.0)	0.34
Largest tumor diameter (cm), median (IQR)	3.0 (2.0, 3.8)	2.5 (2.0, 3.1)	0.20
Outcomes			
Tumor recurrence, n (%)	​	​	**0.001**
No	10 (52.6)	51 (89.5)	​
Yes	9 (47.4)	6 (10.5)	​
Patient status, n (%)	​	​	**0.003**
Deceased	13 (68.4)	16 (28.1)	​
Alive	6 (31.6)	41 (71.9)	​
Propensity score matching criteria			
AFP at listing (ng/mL), median (IQR)	7.1 (2.7, 22.2)	7.9 (4.3, 32.9)	0.22
Total radiographic total tumor diameter at listing (cm), median (IQR)	2.2 (1.3, 4.3)	2.3 (1.2, 5.3)	0.71
Total number of tumors at last scan pre-LT, median (IQR)	1.0 (1.0, 2.0)	1.0 (1.0, 2.0)	0.80

Bold values denote statistical significance (p < 0.05).

AFP, alpha fetoprotein; BMI, body mass index; HCC, hepatocellular carcinoma; IQR, interquartile range; LRT, locoregional therapies; LT, liver transplantation; MASLD, metabolic dysfunction-associated steatotic liver disease; MELD, Model for End-Stage Liver Disease, TACE, transarterial chemoembolization; UCSF, University of California, San Francisco.

Because the patients were thought to have HCC prior to LT based on imaging characteristics, they received neoadjuvant therapies directed at HCC. Most (n = 16, 84.2%) received some type of neoadjuvant therapy (Table 1). TACE was most frequently used (n = 11, 57.9%), followed by sorafenib (n = 5, 26.3%), and radiofrequency ablation (RFA, n = 4, 21.1%, including 1 patient who received both RFA and TACE). Three patients (15.8%) received microwave ablation and two (10.5%) received yttrium-90 (Y90) as neoadjuvant treatment. The 5 patients who received neoadjuvant sorafenib underwent treatment in 2015 or earlier and are included in the count of those who received TACE. Based on pre-LT radiographic measurements, most (n = 16, 84.2%) were within Milan criteria. Two patients (10.5%) were outside Milan but within University of California, San Francisco (USCF) criteria, and one patient (5.3%) was outside UCSF criteria (Table 1).

The median largest tumor size in the HCC-CCA cases was ∼3.0 (IQR, 2.0–3.8); most patients had multifocal disease. Based on pathology results, 7 (36.8%) patients were within Milan, 6 (31.6%) were outside Milan and within UCSF, and 6 (31.6%) were outside USCF criteria. Of the patients who were outside UCSF criteria on explant, 5 patients responded to LRT with tumor size stabilization or reduction. One patient was transplanted urgently and had only hepatic ultrasound pre-LT; thus, this case did not receive neoadjuvant chemotherapy or LRT.

Most patients had T2 tumors (9, 47.4%), based on pathologic findings in the explants (Table 1). One patient (5.3%) had nodal metastases. None of the patients had macrovascular invasion, and 2 (10.5%) patients were found to have microvascular invasion (Supplementary Table S2). Most patients had moderately (n = 9, 47.4%) or poorly (n = 9, 47.4%) differentiated tumors; only one (1.8%) patient had well-differentiated HCC-CCA.

Data on post-LT adjuvant therapy was available for 18 of 19 patients. Of those 18 patients, 13 received adjuvant therapy with most patients receiving gemcitabine- or capecitabine-based therapy (Supplementary Table S3). None of the patients had metastatic disease at transplant.

**FIGURE 1:**
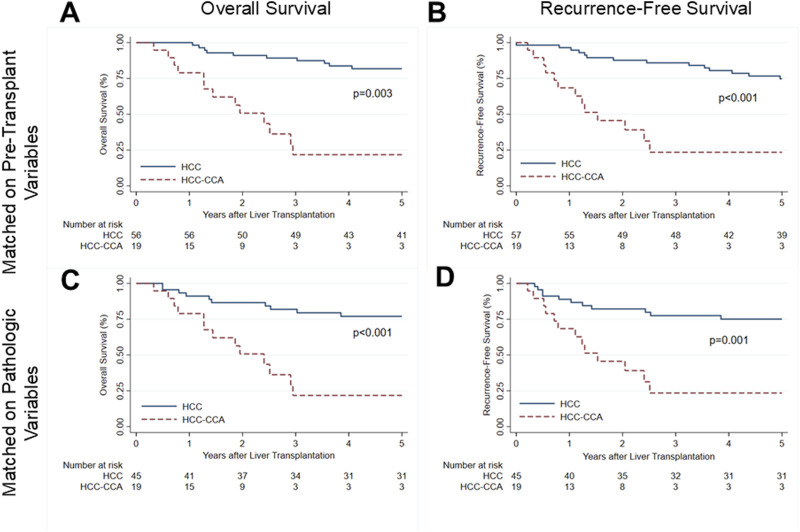
Survival after liver transplantation for patients with mixed hepatocellular carcinoma-cholangiocarcinoma (solid line) and propensity-matched patients with hepatocellular carcinoma (dashed line). **(A)** Overall patient survival and **(B)** recurrence-free survival for mixed tumor patients and patients with HCC matched on pre-transplant characteristics. **(C)** Overall patient survival and **(D)** recurrence-free survival for mixed tumor patients and HCC patients matched on explant pathology characteristics. HCC, hepatocellular carcinoma; HCC-CCA, mixed hepatocellular carcinoma-cholangiocarcinoma.

At a median post-transplant follow-up of 1.95 years, 13 (68.4%) of the 19 patients with HCC-CCA were deceased (Table 1). Nine (47.4%) patients ultimately died from metastatic adenocarcinoma, 2 (10.5%) from cardiac arrest, 1 (5.3%) from multi-system organ failure, and 1 (5.3%) from respiratory failure. Overall survival (OS) rates of patients with mixed tumors were 78.9% at 1 year and 23.8% at 3 and 5 years post-LT (Figure 1A). Recurrence-free survival (RFS) was 68.4% at 1 year and 26.8% at 3 and 5 years after transplant (Figure 1B).

### Pre-Liver Transplantation Matched Hepatocellular Carcinoma Patient Comparison

The pre-LT match included 57 patients with HCC matched to the 19 patients with HCC-CCA based on pre-transplant characteristics. No missing variables occurred in the match. Characteristics of the entire HCC cohort are presented in Supplementary Table S1. Demographically, patients with mixed tumor and HCC alone were similar, and they also had similar indications for LT (all p > 0.05; Table 1; Supplementary Table S2). There were also no differences in severity of illness: laboratory MELD (p = 0.57), listing MELD (p = 0.80), and medical condition at transplant (p = 1.00) were all statistically similar.

Serum tumor markers (AFP, neutrophil-to-leukocyte ratio) at the time of listing and prior to LT were also not significantly different between patients with HCC-CCA and matched patients with HCC (all p > 0.05) when matched on pre-LT features (Table 1). Radiographically, tumor number (p = 0.86) and total tumor diameter at listing (p = 0.71) were similar. Patients with HCC-CCA also received similar types of neoadjuvant therapy (all p > 0.05) apart from microwave ablation (MWA), which occurred more often in HCC-CCA (p = 0.01; Table 1). The number of locoregional therapy treatments were similar between groups (p = 0.70; Table 1). HCC T and N staging based on explant pathology was also similar between patients with HCC-CCA and pre-LT matched HCC alone (T stage, p = 0.30; N stage, p = 0.37; Table 1). Both groups also had similar pathologic estimated tumor necrosis (p = 0.40). Importantly, although pre-LT tumor burden was similar between groups, a significantly greater proportion of patients with HCC-CCA were found to be outside Milan and UCSF criteria on explant, indicative of the true biological disease burden (p = 0.01; Table 1).

Relative to matched patients with HCC, patients with HCC-CCA had significantly lower OS (log-rank: p = 0.003; Figure 1A) and RFS (log-rank: p < 0.001; Figure 1B). By Cox proportional hazards analysis, OS for patients with HCC-CCA had a hazard ratio (HR) of 6.47 (95% CI, 2.87–14.55; p < 0.001) relative to patients with HCC only matched on pre-LT features. Similarly, RFS rates were significantly inferior for patients with HCC-CCA (HR, 5.55; 95% CI, 2.58–11.97; p < 0.001).

### Explant Pathology Matched Hepatocellular Carcinoma Patient Comparison

The second, independent propensity match using explant pathology features (“explant match”) included 45 patients with HCC matched to the core cohort of 19 patients with HCC-CCA. No missing variables occurred in the match. The patients with HCC-CCA had similar demographics as the patients with explant-matched HCC (all p > 0.05; Supplementary Table S4). Etiology of liver disease was not significantly different between the two groups (p = 0.62; Table 2), and they also had similar laboratory MELD scores at transplant (p = 0.58). AFP levels at listing and immediately prior to transplant were not statistically different between patients with HCC-CCA and patients with HCC (p = 0.07 and p = 0.75, respectively; Table 2). Patients with HCC-CCA were more likely to have received neoadjuvant MWA than patients with HCC matched on explant pathology features (15.8% vs. 0%; p = 0.02; Table 2).

**TABLE 2:** Clinical characteristics of liver transplant recipients with mixed hepatocellular carcinoma-cholangiocarcinoma and propensity-matched patients with hepatocellular carcinoma only based on explant pathology variables (“explant match”).

Recipient Characteristics	Mixed Tumor	HCC Explant Match	p-value
	N = 19	N = 45	
Laboratory MELD at transplant, median (IQR)	17.0 (9.0, 29.0)	13.0 (10.0, 25.0)	0.58
List MELD at txp, median (IQR)	29.0 (26.0, 33.0)	29.0 (27.0, 32.0)	0.85
Underlying etiology of liver disease, n (%)	​	​	0.62
Hepatitis C	8 (42.1)	27 (60.0)	​
Hepatitis B	2 (10.5)	2 (4.4)	​
Alcohol-associated liver disease	3 (15.8)	5 (11.1)	​
MASLD or cryptogenic cirrhosis	5 (26.3)	8 (17.8)	​
Other	1 (5.3)	3 (6.7)	​
Waiting time from listing (days), median (IQR)	332.0 (148.0, 806.0)	364.0 (206.0, 571.0)	0.98
Pre-transplant tumor markers			
AFP at listing (ng/mL), median (IQR)	7.1 (2.7, 22.2)	17.2 (4.8, 56.1)	0.07
Last AFP prior to transplant (ng/mL), median (IQR)	5.2 (2.6, 20.9)	7.6 (3.6, 25.1)	0.75
Neutrophil/lymphocyte ratio pre-LT, median (IQR)	3.5 (2.0, 7.2)	4.4 (2.2, 9.2)	0.59
Neoadjuvant therapy			
Neoadjuvant therapy, n (%)			
TACE	11 (57.9)	33 (73.3)	0.25
Radiofrequency ablation	4 (21.1)	13 (28.9)	0.76
Resection	0 (0.0)	3 (6.7)	0.55
Sorafenib	5 (26.3)	17 (37.8)	0.57
Yttrium-90	2 (10.5)	5 (11.1)	1.00
Microwave ablation	3 (15.8)	0 (0.0)	**0.02**
Any neoadjuvant therapy, n (%)	16 (84.2)	41 (91.1)	0.41
Total number of LRT, median (IQR)	2.0 (1.0, 3.0)	2.0 (1.0, 2.0)	0.78
Pre-transplant radiographic tumor characteristics			
Tumor burden classification, n (%)	​	​	0.11
Within Milan	16 (84.2)	25 (55.6)	​
Outside Milan, within UCSF	2 (10.5)	11 (24.4)	​
Outside UCSF	1 (5.3)	9 (20.0)	​
Total radiographic total tumor diameter at listing (cm), median (IQR)	2.2 (1.3, 4.3)	3.5 (2.1, 5.3)	0.09
Total number of tumors at listing, median (IQR)	1.0 (1.0, 2.0)	1.0 (1.0, 2.0)	0.84
Pathologic tumor characteristics			
Tumor burden classification, n (%)	​	​	0.44
Within Milan	7 (36.8)	17 (37.8)	​
Outside Milan, within UCSF	6 (31.6)	8 (17.8)	​
Outside UCSF	6 (31.6)	20 (44.4)	​
HCC necrosis estimate (%), median (IQR)	35.0 (10.0, 80.0)	65.0 (20.0, 95.0)	0.32
Tumor location, n (%)	​	​	0.26
Right lobe	8 (42.1)	27 (60.0)	​
Left lobe	3 (15.8)	4 (8.9)	​
Bilobar	7 (36.8)	14 (31.1)	​
Other	1 (5.3)	0 (0.0)	​
Tumor T stage, n (%)	​	​	0.49
T0	1 (5.3)	1 (2.2)	​
T1s	1 (5.3)	0 (0.0)	​
T1	4 (21.1)	14 (31.1)	​
T2	9 (47.4)	20 (44.4)	​
T3a	2 (10.5)	8 (17.8)	​
T3b	1 (5.3)	1 (2.2)	​
T4	1 (5.3)	1 (2.2)	​
Tumor N Stage, n (%)	​	​	0.36
N0	8 (42.1)	27 (60.0)	​
N1	0 (0.0)	1 (2.2)	​
NX	11 (57.9)	17 (37.8)	​
Microvascular invasion, n (%)	2 (10.5)	1 (2.2)	0.21
Total number of tumors (pathology), median (IQR)	2.0 (2.0, 4.0)	2.0 (1.0, 4.0)	0.87
Largest tumor diameter (cm), median (IQR)	3.0 (2.0, 3.8)	3.5 (2.7, 4.7)	0.08
Outcomes			
Tumor recurrence, n (%)	​	​	**0.01**
No	10 (52.6)	38 (84.4)	​
Yes	9 (47.4)	7 (15.6)	​
Patient status, n (%)	​	​	**0.005**
Deceased	13 (68.4)	13 (28.9)	​
Alive	6 (31.6)	32 (71.1)	​
Total intraoperative PRBC (units), median (IQR)	4.0 (2.0, 10.0)	5.0 (2.0, 6.0)	0.99
Propensity score matching criteria			
Pathologic total tumor diameter (cm), median (IQR)	5.6 (3.5, 7.3)	6.0 (4.1, 11.0)	0.83
Pathologic differentiation, n (%)	​	​	0.68
Well	1 (5.3)	3 (6.7)	​
Moderate	9 (47.4)	27 (60.0)	​
Poor	9 (47.4)	15 (33.3)	​
Any vascular invasion, n (%)	​	​	0.21
No	17 (89.5)	44 (97.8)	​
Yes	2 (10.5)	1 (2.2)	​

Bold values denote statistical significance (p < 0.05).

AFP, alpha fetoprotein; BMI, body mass index; HCC, hepatocellular carcinoma; IQR, interquartile range; LRT, locoregional therapies; LT, liver transplantation; MASLD, metabolic dysfunction-associated steatotic liver disease; MELD, Model for End-Stage Liver Disease, TACE, transarterial chemoembolization; UCSF, University of California, San Francisco.

Disease burden was similar between HCC-CCA and HCC alone in the “explant match” group. The proportion of patients within or outside Milan or USCF criteria were similar between groups (p = 0.44). Pre-transplant radiographic lesion number (p = 0.84) and total tumor diameter (p = 0.09) were not statistically different. Total tumor number (p = 0.87) and tumor diameter (p = 0.08) on explant were also statistically comparable. T (p = 0.49) and N (p = 0.36) staging were similar between groups (Table 2). Microvascular invasion rates were similar among HCC-CCA (10.5%) and HCC alone in the “explant match” group (2.2%; p = 0.21).

Mixed tumor cases had significantly lower OS (log-rank: p < 0.001; Figure 1C) and RFS (log-rank: p = 0.001; Figure 1D) rates than those with HCC alone matched on explant features. In univariable Cox proportional hazards analysis, both OS (HR, 4.67; 95% CI, 2.07–10.53; p = 0.0003) and RFS (HR, 3.65; 95% CI, 1.70–7.82; p = 0.001) were significantly inferior for patients with HCC-CCA.

## Discussion

Patients with HCC-CCA had significantly worse OS and RFS than HCC, despite similar clinical features. This reflects the aggressive biology and challenges of pre-LT diagnosis. These comparisons identified patients with HCC with similar features that inform pre-LT clinical decision making and that approximate actual pathologic risk, respectively. Unfortunately, the mixed tumor patients had low AFP and CA19-9 levels and were radiographically similar to patients with HCC alone, making correct pre-LT diagnosis difficult. Like many other studies, the patients with mixed tumor in this cohort were originally diagnosed with HCC and thus received HCC-directed neoadjuvant therapy. It is likely that the CCA component of the mixed HCC-CCA lesions did not receive appropriate neoadjuvant systemic treatment, such as standard chemotherapy regimens for CCA. All patients with intrahepatic CCA considered for LT at our institution receive neoadjuvant gemcitabine and cisplatin as bridging and downstaging therapy, which is also used to inform patient selection [17]. It was not possible to follow the standard protocol in patients who were determined to have HCC-CCA only at the time of examination of the liver explant. These patients did receive CCA-focused adjuvant therapy. Due to the small sample size, we are unable to make conclusions regarding the efficacy of one adjuvant therapy over another, and the adjuvant regimens remain hypothesis-generating only.

The propensity match analysis provides insight into the clinical presentation and biology of HCC-CCA compared to HCC with similar pre-LT (Match 1) and post-LT (Match 2) characteristics. Matching on a small subset of prognostic variables allows us to better understand differences in mixed tumors and HCC when controlling for tumor burden. In both analyses, patients with HCC-CCA were more likely to have received neoadjuvant MWA. MWA has been shown to be an effective treatment in both HCC [18] and iCCA [19], and single-center studies have demonstrated its efficacy as a bridging [20, 21] and downstaging therapy [22]. Thus, it is unlikely that the use of MWA significantly affected outcomes. Patients matched on pre-LT characteristics (Match 1) were more likely to be outside Milan criteria on explant, highlighting the difficulty of radiologic staging in biologically aggressive tumors. This result highlights the difficulty of estimating tumor burden pre-LT in patients whose tumors have aggressive biology, such as HCC-CCA. It is interesting that 9 of the HCC-CCA patients with tumors inside Milan criteria radiologically pre-LT were outside Milan on explant pathology. Only 2 patients were outside Milan criteria due to lymphovascular invasion, which is very difficult to detect radiologically.

The 3- and 5-year OS rates for the cohort of 19 patients with HCC-CCA described here were much lower than other papers have reported (Table 3). Most patients with mixed tumors were thought to be within Milan criteria pre-LT (16, 84.2%), demonstrating that poor outcomes can occur even with a small tumor burden. Other papers have also shown that patients with HCC-CCA have worse outcomes than matched patients with HCC alone [13, 23–25], although some centers have reported similar survival rates [8, 9, 26, 27]. The OS rates reported here are also much lower than what our own center has demonstrated for patients with large HCC tumor burden (beyond UCSF criteria) [28], and for patients with intrahepatic CCA [17]. Penzkofer and colleagues noted that outcomes seemed to be more strongly associated with the CCA component of the mixed tumors [11]; our data also support this conclusion.

**TABLE 3:** Summary of survival outcomes of literature reports of patients undergoing liver transplantation for combined hepatocellular-cholangiocarcinoma.

Paper	Year	n	Data source	Dates	1y OS (%)	3y OS (%)	5y OS (%)
Present study	​	19	Texas, US (single center)	2008–2022	78.9	23.8	23.8
Panjala et al.	2010	12	Florida, US (single center)	1998–2008	79	66	16
Groeschl et al.	2013	19	SEER database (US)	1973–2008	89	48	​
Sapisochin et al.	2014	15	Spain (multicenter)	2000–2010	93	78	78
Vilchez et al.	2016	94	UNOS database	1994–2013	82	47	40
Jung et al.	2017	32	Seoul, South Korea (single center)	2005–2014	84.4	73.1	65.8
Antwi et al.	2018	19	Florida, US (single center)	2001–2016	84	74	​
Lunsford et al.	2018	12	California, US (single center)	1984–2015	75	54	42
Li et al.	2019	301	Meta-analysis	2000–2018	​	​	41
Spolverato et al.	2019	220	National cancer database	2004–2015	​	​	52.6
Dageforde et al.	2021	99	US consortium (12 centers)	2009–2017	89.1^a^	77.1^a^	70.1^a^
Jaradat et al.	2021	19	Germany, Turkey, Jordan (multi-center)	2001–2018	57.1	38.1	​
Brandão et al.	2022	7	Brazil (single center)	1997–2019	85.7	​	54.1
Chen et al.	2022	60	SEER database (US)	2004–2015	86.7	68.3	56.6
Anilir et al.	2023	17	Turkey (single center)	2004–2019	80.2	57.3	66.7
Garcia-Moreno et al.	2023	6	Spain (single center)	2006–2019	83.3	66.7	66.7
Mi et al.	2023	49	SEER database (US)	2000–2018	86.2	72.4	60.3
Penzkofer et al.	2023	6	Germany (single center)	2008–2020	100	100	80
Kim et al.	2023	111	Korea (multicenter)	2000–2018	84.4	63.8	​

^a^ Patients within Milan criteria.

This study highlights the need for unique biomarkers, whether blood, tissue, or imaging, to distinguish HCC from HCC-CCA. Other studies of LT recipients have shown that CCA-specific biomarkers like CA19-9 were similar between patients with pure HCC and patients with mixed tumors [13]. Given that patients with mixed tumors have reduced expected post-LT survival, better methods for diagnosing HCC-CCA pre-LT are needed, particularly as the number of patients undergoing LT for oncologic indications increases. Patients whose lesions are identified as liver imaging reporting and data system (LIRADS)-M, presumed to have likely or definite malignancy, or imaging characteristics not typical of HCC, should undergo image-guided biopsy for definitive diagnosis. If a patient is confirmed to have HCC-CCA, centers should obtain genetic profiling/next-generation sequencing data to optimize tailoring treatment. Such genomic data can help guide physicians in selecting neoadjuvant systemic options that will treat both components (hepatocellular and adenocarcinoma) of the cancer. Given poor outcomes and high rates of recurrence, institution-based protocols are necessary for treating these aggressive cancers, considering the dearth of data in post-transplant outcomes in patients with HCC-CCA.

This study is limited by its retrospective nature and because it only incorporates patients from a single center. Additionally, very few patients underwent LT for HCC-CCA during the study period, limiting the sample size of this cohort, particularly at longer follow-up (>3 years post-LT) when many patients had died. Thus, the small number of patients could reduce the confidence in long-term follow up outcomes. Although our institution is in a region with high racial and ethnic diversity, the results presented here may not accurately reflect the experiences at other centers. There might be residual confounding arising from unmeasured patients’ attributes. Despite these limitations, this study has several strengths. To our knowledge, this manuscript presents the largest propensity score matching analysis of patients undergoing LT for HCC-CCA vs. HCC using multiple variables to select patients with similar disease burden. Thus, the conclusions drawn here provide important insight into the surgical treatment of patients with HCC-CCA. Another major strength of this cohort is the diverse patient population at our center. Although most patients with HCC-CCA were non-Hispanic White, this work still provides an accurate representation of the incidence of HCC-CCA in a diverse transplant population.

This manuscript provides a propensity-score matched comparison utilizing granular center medical records of patients receiving LT from deceased donors for HCC where incidental HCC-CCA occurred. Although LT can offer superior OS relative to resection [7, 9, 11], the study showed that survival is still much lower for patients with HCC-CCA than for HCC, the most common oncologic indication for LT. Given that most patients with HCC-CCA are diagnosed incidentally after transplant and are associated with inferior outcomes, allocation policy may need to weigh whether LT for HCC-CCA is justified without better selection tools and treatment options. Accurate pre-LT diagnosis may have allowed patients with HCC-CCA to receive adequate neoadjuvant treatment for the CCA component of the tumor, potentially improving outcomes. Better screening techniques are needed to identify patients with these rare tumors pre-transplant to ensure they receive the most appropriate treatment possible. Liquid biopsy and next-generation sequencing show promise in helping to accurately distinguish HCC-CCA from HCC [29]. Given the improved survival after LT for HCC-CCA relative to resection, increasing utilization of machine perfusion [30] and extended criteria donor grafts [31–34] may allow greater expansion of LT to well-selected patients.

## Data Availability

The data analyzed in this study is subject to the following licenses/restrictions: Individual privacy is a concern when reporting on rare conditions. Therefore, the data are not available to be released. Requests to access these datasets should be directed to rmghobrial@houstonmethodist.org.
