# Supplementary material for: Incidental Combined Hepatocellular-Cholangiocarcinoma in Liver Transplant Recipients: A Matched Cohort Study
Source: Transpl Int. 2026 Jan 28;38:15298. doi: 10.3389/ti.2025.15298 (PMC12890706; doi:10.3389/ti.2025.15298)
Supplement: Supplementary file 1 [file DataSheet1.pdf]

Supplementary Material

Supplementary Table S1. Demographic, clinical, and donor characteristics for liver transplant recipients with mixed hepatocellular carcinoma-cholangiocarcinoma or hepatocellular carcinoma.

	HCC N=240	Mixed Tumor N=19	p-value
**Recipient Characteristics**			
Age (years), median (IQR)	61.0 (57.0, 66.0)	65.2 (61.1, 69.5)	**0.045**
Sex, n (%)			0.60
Male	70 (29.2)	4 (21.1)	
Female	170 (70.8)	15 (78.9)	
Race/ethnicity, n (%)			0.35
White	165 (69.0)	14 (73.7)	
Black	24 (10.0)	0 (0.0)	
Hispanic	38 (15.9)	3 (15.8)	
Asian	12 (5.0)	2 (10.5)	
BMI at LT (kg/m^2), median (IQR)	27.6 (24.5, 32.3)	29.6 (24.8, 33.9)	0.51
Laboratory MELD at transplant, median (IQR)	13.0 (9.0, 20.5)	17.0 (9.0, 29.0)	0.30
Underlying etiology of liver disease, n (%)			0.08
Hepatitis C	162 (67.5)	8 (42.1)	
Hepatitis B	8 (3.3)	2 (10.5)	
Alcohol-associated liver disease	23 (9.6)	3 (15.8)	
MASLD or cryptogenic cirrhosis	34 (14.2)	5 (26.3)	
Other	13 (5.4)	1 (5.3)	
Waiting time from listing (days), median (IQR)	340.5 (190.0, 520.0)	332.0 (148.0, 806.0)	0.60
History of diabetes mellitus, n (%)	102 (42.5)	13 (68.4)	**0.03**
History of hypertension, n (%)	146 (60.8)	16 (84.2)	**0.049**
Medical condition at LT, n (%)			1.00
Home	186 (77.5)	15 (78.9)	
Floor	23 (9.6)	2 (10.5)	
ICU	31 (12.9)	2 (10.5)	
**Pre-transplant tumor markers**			
AFP at listing (ng/mL), median (IQR)	9.6 (5.1, 35.1)	7.1 (2.7, 22.2)	0.10
Last AFP prior to transplant (ng/mL), median (IQR)	6.7 (3.7, 21.0)	5.2 (2.6, 20.9)	0.61
Maximum AFP pre-LT (ng/mL), median (IQR)	15.4 (6.4, 64.6)	12.6 (4.8, 37.4)	0.47
Neutrophil/lymphocyte ratio pre-LT, median (IQR)	4.5 (2.3, 11.8)	3.5 (2.0, 7.2)	0.24
**Neoadjuvant therapy**			
Neoadjuvant therapy, n (%)			
TACE	183 (76.3)	11 (57.9)	0.10
Radiofrequency ablation	67 (27.9)	4 (21.1)	0.60
Resection	11 (4.6)	0 (0.0)	1

	HCC N=240	Mixed Tumor N=19	p-value
**Recipient Characteristics**			
Sorafenib	58 (24.2)	5 (26.3)	0.79
Yttrium-90	23 (9.6)	2 (10.5)	0.70
Microwave ablation	1 (0.4)	3 (15.8)	**0.001**
Any neoadjuvant therapy, n (%)	216 (90.0)	16 (84.2)	0.43
Total number of LRT, median (IQR)	1.0 (1.0, 2.0)	2.0 (1.0, 3.0)	0.21
**Pre-transplant radiographic tumor characteristics**			
Tumor burden classification, n (%)			0.72
Within Milan	178 (74.5)	16 (84.2)	
Outside Milan, Within UCSF	29 (12.1)	2 (10.5)	
Outside UCSF	32 (13.4)	1 (5.3)	
Total radiographic tumor size at listing (cm), median (IQR)	3.0 (1.9, 4.6)	2.2 (1.3, 4.3)	0.24
Total number of tumors at listing, median (IQR)	1.0 (1.0, 2.0)	1.0 (1.0, 2.0)	0.72
Total number of tumors at last scan pre-LT, median (IQR)	1.0 (0.0, 1.0)	1.0 (1.0, 2.0)	**0.008**
**Pathologic tumor characteristics**			
Tumor burden classification, n (%)			**0.03**
Within Milan	145 (60.4)	7 (36.8)	
Outside Milan, Within UCSF	26 (10.8)	6 (31.6)	
Outside UCSF	69 (28.7)	6 (31.6)	
HCC necrosis estimate (%), median (IQR)	77.5 (30.0, 96.0)	35.0 (10.0, 80.0)	0.05
Tumor Location, n (%)			**0.046**
Right Lobe	105 (63.3)	8 (42.1)	
Left Lobe	18 (10.8)	3 (15.8)	
Bilobar	43 (25.9)	7 (36.8)	
Other	0 (0.0)	1 (5.3)	
Tumor T Stage, n (%)			0.13
T0	25 (15.2)	1 (5.3)	
T1s	1 (0.6)	1 (5.3)	
T1	52 (31.5)	4 (21.1)	
T2	70 (42.4)	9 (47.4)	
T3a	12 (7.3)	2 (10.5)	
T3b	3 (1.8)	1 (5.3)	
T4	2 (1.2)	1 (5.3)	
Tumor N Stage, n (%)			0.40
N0	92 (56.1)	8 (42.1)	
N1	1 (0.6)	0 (0.0)	
NX	71 (43.3)	11 (57.9)	
Multifocal, n (%)	127 (52.9)	16 (84.2)	**0.008**
Microvascular invasion, n (%)	10 (6.0)	2 (10.5)	0.35

	HCC<br>N=240	Mixed Tumor<br>N=19	p-value
**Recipient Characteristics**			
Macrovascular invasion, n (%)	6 (3.6)	0 (0.0)	1.00
Any vascular invasion, n (%)			0.21
Present (yes)	13 (7.8)	2 (10.5)	
Absent (no)	154 (92.2)	17 (89.5)	
Pathologic differentiation, n (%)			**<0.001**
Well	69 (35.0)	1 (5.3)	
Moderate	110 (55.8)	9 (47.4)	
Poor	18 (9.1)	9 (47.4)	
Total number of tumors (pathology), median (IQR)	2.0 (1.0, 3.0)	2.0 (2.0, 4.0)	0.08
Pathologic total tumor diameter (cm), median (IQR)	4.0 (2.5, 7.0)	5.6 (3.5, 7.3)	**0.03**
Largest tumor diameter (cm), median (IQR)	2.8 (2.0, 4.0)	3.0 (2.0, 3.8)	0.5
**Donor characteristics**			
Donor age (years), median (IQR)	36.0 (24.0, 48.5)	37.0 (28.0, 54.0)	0.24
Donor sex, n (%)			0.34
Male	105 (44.3)	6 (31.6)	
Female	132 (55.7)	13 (68.4)	
Donor type, n (%)			0.64
Donation after brain death	223 (92.9)	17 (89.5)	
Donation after circulatory death	17 (7.1)	2 (10.5)	
Cold ischemia time (hours), median (IQR)	6.5 (5.0, 8.2)	6.4 (4.5, 8.5)	0.9
**Outcomes**			
Tumor recurrence, n (%)			**<0.001**
No	214 (89.2)	10 (52.6)	
Yes	26 (10.8)	9 (47.4)	
Patient status, n (%)			**0.001**
Deceased	72 (30.0)	13 (68.4)	
Alive	168 (70.0)	6 (31.6)	
Total intraoperative PRBC (units), median (IQR)	5.0 (2.0, 8.0)	4.0 (2.0, 10.0)	0.85

Supplementary Table S2. Demographic, clinical, and donor characteristics for liver transplant recipients with mixed hepatocellular carcinoma-cholangiocarcinoma and propensity-matched patients with hepatocellular carcinoma only based on pre-transplant variables ("pre-LT match").

	Mixed Tumor N=19	HCC Pre-LT Match N=57	p-value
**Recipient Characteristics**			
Age (years), median (IQR)	65.2 (61.1, 69.5)	62.0 (57.0, 67.0)	0.12
Sex, n (%)			0.77
Male	4 (21.1)	15 (26.3)	
Female	15 (78.9)	42 (73.7)	
Race/ethnicity, n (%)			0.76
White	14 (73.7)	39 (68.4)	
Black	0 (0.0)	4 (7.0)	
Hispanic	3 (15.8)	10 (17.5)	
Asian	2 (10.5)	4 (7.0)	
BMI at LT (kg/m^2), median (IQR)	29.6 (24.8, 33.9)	27.3 (23.9, 32.7)	0.64
Laboratory MELD at transplant, median (IQR)	17.0 (9.0, 29.0)	13.0 (10.0, 19.0)	0.57
Underlying etiology of liver disease, n (%)			0.19
Hepatitis C	8 (42.1)	39 (68.4)	
Hepatitis B	2 (10.5)	3 (5.3)	
Alcohol-associated liver disease	3 (15.8)	7 (12.3)	
MASLD or cryptogenic cirrhosis	5 (26.3)	7 (12.3)	
Other	1 (5.3)	1 (1.8)	
Waiting time from listing (days), median (IQR)	332.0 (148.0, 806.0)	346.0 (190.0, 525.0)	0.67
History of diabetes mellitus, n (%)	13 (68.4)	27 (47.4)	0.18
History of hypertension, n (%)	16 (84.2)	34 (59.6)	0.06
Medical condition at LT, n (%)			1.00
Home	15 (78.9)	43 (75.4)	
Floor	2 (10.5)	6 (10.5)	
ICU	2 (10.5)	8 (14.0)	
**Pre-transplant tumor markers**			
AFP at listing (ng/mL), median (IQR)	7.1 (2.7, 22.2)	7.9 (4.3, 32.9)	0.22
Last AFP prior to transplant (ng/mL), median (IQR)	5.2 (2.6, 20.9)	6.6 (3.6, 27.3)	0.63
Maximum AFP pre-LT (ng/mL), median (IQR)	12.6 (4.8, 37.4)	11.9 (6.5, 88.1)	0.65
Neutrophil/lymphocyte ratio pre-LT, median (IQR)	3.5 (2.0, 7.2)	5.8 (2.7, 17.9)	0.15
**Neoadjuvant therapy**			
Neoadjuvant therapy, n (%)			
TACE	11 (57.9)	43 (75.4)	0.16
Radiofrequency ablation	4 (21.1)	13 (22.8)	1.00
Resection	0 (0.0)	2 (3.5)	1.00
Sorafenib	5 (26.3)	14 (24.6)	1.00
Yttrium-90	2 (10.5)	3 (5.3)	0.59

	Mixed Tumor N=19	HCC Pre-LT Match N=57	p-value
**Recipient Characteristics**			
Microwave ablation	3 (15.8)	0 (0.0)	**0.01**
Any neoadjuvant therapy, n (%)	16 (84.2)	50 (87.7)	0.70
Total number of LRT, median (IQR)	2.0 (1.0, 3.0)	1.0 (1.0, 2.0)	0.09
**Pre-transplant radiographic tumor characteristics**			
Tumor burden classification, n (%)			0.54
Within Milan	16 (84.2)	45 (78.9)	
Outside Milan, Within UCSF	2 (10.5)	4 (7.0)	
Outside UCSF	1 (5.3)	8 (14.0)	
Total radiographic total tumor diameter at listing (cm), median (IQR)	2.2 (1.3, 4.3)	2.3 (1.2, 5.3)	0.71
Total number of tumors at listing, median (IQR)	1.0 (1.0, 2.0)	1.0 (1.0, 2.0)	0.86
Total number of tumors at last scan pre-LT, median (IQR)	1.0 (1.0, 2.0)	1.0 (1.0, 2.0)	0.80
**Pathologic tumor characteristics**			
Tumor burden classification, n (%)			**0.01**
Within Milan	7 (36.8)	38 (66.7)	
Outside Milan, Within UCSF	6 (31.6)	4 (7.0)	
Outside UCSF	6 (31.6)	15 (26.3)	
HCC necrosis estimate (%), median (IQR)	35.0 (10.0, 80.0)	60.0 (22.0, 95.0)	0.40
Tumor Location, n (%)			0.22
Right Lobe	8 (42.1)	22 (62.9)	
Left Lobe	3 (15.8)	2 (5.7)	
Bilobar	7 (36.8)	11 (31.4)	
Other	1 (5.3)	0 (0.0)	
Tumor T Stage, n (%)			0.30
T0	1 (5.3)	1 (2.8)	
T1s	1 (5.3)	0 (0.0)	
T1	4 (21.1)	15 (41.7)	
T2	9 (47.4)	17 (47.2)	
T3a	2 (10.5)	2 (5.6)	
T3b	1 (5.3)	1 (2.8)	
T4	1 (5.3)	0 (0.0)	
Tumor N Stage, n (%)			0.37
N0	8 (42.1)	21 (58.3)	
N1	0 (0.0)	1 (2.8)	
NX	11 (57.9)	14 (38.9)	
Multifocal, n (%)	16 (84.2)	36 (63.2)	0.15
Microvascular invasion, n (%)	2 (10.5)	2 (5.6)	0.60
Macrovascular invasion, n (%)	0 (0.0)	0 (0.0)	
Total number of tumors (pathology), median (IQR)	2.0 (2.0, 4.0)	2.0 (1.0, 3.0)	0.34

	Mixed Tumor<br>N=19	HCC Pre-LT Match<br>N=57	p-value
**Recipient Characteristics**			
Largest tumor diameter (cm), median (IQR)	3.0 (2.0, 3.8)	2.5 (2.0, 3.1)	0.20
**Donor characteristics**			
Donor age (years), median (IQR)	37.0 (28.0, 54.0)	37.0 (23.0, 50.0)	0.36
Donor sex, n (%)			0.29
Male	6 (31.6)	26 (47.3)	
Female	13 (68.4)	29 (52.7)	
Donor type, n (%)			1.00
Donation after brain death	17 (89.5)	52 (91.2)	
Donation after circulatory death	2 (10.5)	5 (8.8)	
Cold ischemia time (hours), median (IQR)	6.4 (4.5, 8.5)	6.4 (5.0, 8.2)	0.95
**Outcomes**			
Tumor recurrence, n (%)			**0.001**
No	10 (52.6)	51 (89.5)	
Yes	9 (47.4)	6 (10.5)	
Patient status, n (%)			**0.003**
Deceased	13 (68.4)	16 (28.1)	
Alive	6 (31.6)	41 (71.9)	
Total intraoperative PRBC (units), median (IQR)	4.0 (2.0, 10.0)	5.0 (3.0, 8.0)	0.63

Bold values denote p<0.05.

BMI, body mass index; ICU, intensive care unit; LT, liver transplantation; MASLD, metabolic dysfunction-associated steatotic liver disease; MELD, Model for End-Stage Liver Disease; PRBC, packed red blood cells

Supplemental Table S3. Available adjuvant therapy information for incidentally diagnosed HCC-CCA patients who underwent LT (n=13)

Adjuvant therapy	Recipients, n (%)
Gemcitabine and Oxaliplatin	4 (30.8)
Capecitabine	4 (30.8)
Gemcitabine and Cisplatin	2 (15.4)
Capecitabine, Gemcitabine, and Oxaliplatin	1 (7.7)
FOLFIRI	1 (7.7)
FOLFOX	1 (7.7)

Supplementary Table S4. Demographic, clinical, and donor characteristics for liver transplant recipients with mixed hepatocellular carcinoma-cholangiocarcinoma and propensity-matched patients with hepatocellular carcinoma only based on pathologic tumor characteristics on explant ("Explant Match").

	Mixed Tumor<br>N=19	HCC Explant Match<br>N=45	p-value
**Recipient Characteristics**			
Age (years), median (IQR)	65.2 (61.1, 69.5)	62.0 (57.0, 68.0)	0.25
Sex, n (%)			0.76
Male	4 (21.1)	13 (28.9)	
Female	15 (78.9)	32 (71.1)	
Race/ethnicity, n (%)			0.49
White	14 (73.7)	30 (66.7)	
Black	0 (0.0)	4 (8.9)	
Hispanic	3 (15.8)	9 (20.0)	
Asian	2 (10.5)	2 (4.4)	
BMI at LT (kg/m^2), median (IQR)	29.6 (24.8, 33.9)	26.0 (22.8, 32.1)	0.21
Laboratory MELD at transplant, median (IQR)	17.0 (9.0, 29.0)	13.0 (10.0, 25.0)	0.58
Underlying etiology of liver disease, n (%)			0.62
Hepatitis C	8 (42.1)	27 (60.0)	
Hepatitis B	2 (10.5)	2 (4.4)	
Alcohol-associated liver disease	3 (15.8)	5 (11.1)	
MASLD or cryptogenic cirrhosis	5 (26.3)	8 (17.8)	
Other	1 (5.3)	3 (6.7)	
Waiting time from listing (days), median (IQR)	332.0 (148.0, 806.0)	364.0 (206.0, 571.0)	0.98
History of diabetes mellitus, n (%)	13 (68.4)	24 (53.3)	0.41
History of hypertension, n (%)	16 (84.2)	28 (62.2)	0.14
Medical condition at LT, n (%)			1.00
Home	15 (78.9)	34 (75.6)	
Floor	2 (10.5)	5 (11.1)	
ICU	2 (10.5)	6 (13.3)	
**Pre-transplant tumor markers**			
AFP at listing (ng/mL), median (IQR)	7.1 (2.7, 22.2)	17.2 (4.8, 56.1)	0.07
Last AFP prior to transplant (ng/mL), median (IQR)	5.2 (2.6, 20.9)	7.6 (3.6, 25.1)	0.75
Maximum AFP pre-LT (ng/mL), median (IQR)	12.6 (4.8, 37.4)	35.8 (7.3, 101.3)	0.16
Neutrophil/lymphocyte ratio pre-LT, median (IQR)	3.5 (2.0, 7.2)	4.4 (2.2, 9.2)	0.59
**Neoadjuvant therapy**			
Neoadjuvant therapy, n (%)			
TACE	11 (57.9)	33 (73.3)	0.25
Radiofrequency ablation	4 (21.1)	13 (28.9)	0.76
Resection	0 (0.0)	3 (6.7)	0.55

	Mixed Tumor<br><br>N=19	HCC Explant Match<br>N=45	p-value
**Recipient Characteristics**			
Sorafenib	5 (26.3)	17 (37.8)	0.57
Yttrium-90	2 (10.5)	5 (11.1)	1.00
Microwave ablation	3 (15.8)	0 (0.0)	**0.02**
Any neoadjuvant therapy, n (%)	16 (84.2)	41 (91.1)	0.41
Total number of LRT, median (IQR)	2.0 (1.0, 3.0)	2.0 (1.0, 2.0)	0.78
**Pre-transplant radiographic tumor characteristics**			
Tumor burden classification, n (%)			0.11
Within Milan	16 (84.2)	25 (55.6)	
Outside Milan, Within UCSF	2 (10.5)	11 (24.4)	
Outside UCSF	1 (5.3)	9 (20.0)	
Total radiographic total tumor diameter at listing (cm), median (IQR)	2.2 (1.3, 4.3)	3.5 (2.1, 5.3)	0.09
Total number of tumors at listing, median (IQR)	1.0 (1.0, 2.0)	1.0 (1.0, 2.0)	0.84
Total number of tumors at last scan pre-LT, median (IQR)	1.0 (1.0, 2.0)	1.0 (0.0, 2.0)	0.09
**Pathologic tumor characteristics**			
Tumor burden classification, n (%)			0.44
Within Milan	7 (36.8)	17 (37.8)	
Outside Milan, Within UCSF	6 (31.6)	8 (17.8)	
Outside UCSF	6 (31.6)	20 (44.4)	
HCC necrosis estimate (%), median (IQR)	35.0 (10.0, 80.0)	65.0 (20.0, 95.0)	0.32
Tumor Location, n (%)			0.26
Right Lobe	8 (42.1)	27 (60.0)	
Left Lobe	3 (15.8)	4 (8.9)	
Bilobar	7 (36.8)	14 (31.1)	
Other	1 (5.3)	0 (0.0)	
Tumor T Stage, n (%)			0.49
T0	1 (5.3)	1 (2.2)	
T1s	1 (5.3)	0 (0.0)	
T1	4 (21.1)	14 (31.1)	
T2	9 (47.4)	20 (44.4)	
T3a	2 (10.5)	8 (17.8)	
T3b	1 (5.3)	1 (2.2)	
T4	1 (5.3)	1 (2.2)	
Tumor N Stage, n (%)			0.36
N0	8 (42.1)	27 (60.0)	
N1	0 (0.0)	1 (2.2)	
NX	11 (57.9)	17 (37.8)	
Multifocal, n (%)	16 (84.2)	30 (66.7)	0.23
Microvascular invasion, n (%)	2 (10.5)	1 (2.2)	0.21

	Mixed Tumor<br><br>N=19	HCC Explant Match<br>N=45	p-value
**Recipient Characteristics**			
Total number of tumors (pathology), median (IQR)	2.0 (2.0, 4.0)	2.0 (1.0, 4.0)	0.87
Largest tumor diameter (cm), median (IQR)	3.0 (2.0, 3.8)	3.5 (2.7, 4.7)	0.08
**Donor characteristics**			
Donor age (years), median (IQR)	37.0 (28.0, 54.0)	34.0 (22.0, 49.0)	0.27
Donor sex, n (%)			1.00
Male	6 (31.6)	15 (33.3)	
Female	13 (68.4)	30 (66.7)	
Donor type, n (%)			1.00
Donation after brain death	17 (89.5)	41 (91.1)	
Donation after circulatory death	2 (10.5)	4 (8.9)	
Cold ischemia time (hours), median (IQR)	6.4 (4.5, 8.5)	6.5 (5.0, 8.1)	0.79
**Outcomes**			
Tumor recurrence, n (%)			**0.01**
No	10 (52.6)	38 (84.4)	
Yes	9 (47.4)	7 (15.6)	
Patient status, n (%)			**0.005**
Deceased	13 (68.4)	13 (28.9)	
Alive	6 (31.6)	32 (71.1)	
Total intraoperative PRBC (units), median (IQR)	4.0 (2.0, 10.0)	5.0 (2.0, 6.0)	0.99

Bold values denote p<0.05.

BMI, body mass index; ICU, intensive care unit; LT, liver transplantation; MASLD, metabolic dysfunction-associated steatotic liver disease; MELD, Model for End-Stage Liver Disease; PRBC, packed red blood cells
